# Fluid-structure-acoustic interactions in an *ex vivo* porcine phonation model

**DOI:** 10.1121/10.0003602

**Published:** 2021-03-10

**Authors:** Marion Semmler, David A. Berry, Anne Schützenberger, Michael Döllinger

**Affiliations:** 1 Division of Phoniatrics and Pediatric Audiology at the Department of Otorhinolaryngology, Head and Neck Surgery, University Hospital Erlangen, Medical School at Friedrich-Alexander-Universität Erlangen-Nürnberg, Waldstrasse 1, 91054 Erlangen, Germany; 2 Laryngeal Dynamics Laboratory, Department of Head and Neck Surgery, David Geffen School of Medicine, UCLA, Los Angeles, California 90024, USA

## Abstract

In the clinic, many diagnostic and therapeutic procedures focus on the oscillation patterns of the vocal folds (VF). Dynamic characteristics of the VFs, such as symmetry, periodicity, and full glottal closure, are considered essential features for healthy phonation. However, the relevance of these individual factors in the complex interaction between the airflow, laryngeal structures, and the resulting acoustics has not yet been quantified. Sustained phonation was induced in nine excised porcine larynges without vocal tract (supraglottal structures had been removed above the ventricular folds). The multimodal setup was designed to simultaneously control and monitor key aspects of phonation in the three essential parts of the larynx. More specifically, measurements will comprise (1) the subglottal pressure signal, (2) high-speed recordings in the glottal plane, and (3) the acoustic signal in the supraglottal region. The automated setup regulates glottal airflow, asymmetric arytenoid adduction, and the pre-phonatory glottal gap. Statistical analysis revealed a beneficial influence of VF periodicity and glottal closure on the signal quality of the subglottal pressure and the supraglottal acoustics, whereas VF symmetry only had a negligible influence. Strong correlations were found between the subglottal and supraglottal signal quality, with significant improvement of the acoustic quality for high levels of periodicity and glottal closure.

## I. INTRODUCTION

Human phonation is not exclusive, but certainly exceptional among mammals.^1^ The underlying mechanism is a highly complex and multifactorial process, which is not yet fully understood, as it involves a large number of variables that interact and depend on each other.^2–4^ The airflow from the lungs excites the primary signal in the larynx resulting from self-sustained oscillations of the vocal folds. The position, shape, and stiffness of their soft and deformable mucosal tissue are controlled by cartilages and muscle tension. Further modulation of the primary signal in the vocal tract (oral and nasal cavity) results in a complex acoustic signal, which exhibits various aspects (i.e., pitch, loudness, noise, tonality) and thereby enables diverse and nuanced expression of humans.^5^ In contrast to the source-filter theory formulated by Fant,^6^ the myoelastic-aerodynamic theory of voice production even allows for interactions between vocal fold vibrations and the subglottal environment in addition to the supraglottal environment.^7,8^ The large degree of variability in each medium and numerous dependencies within the so-called fluid-structure-acoustic interaction (i.e., airflow-vocal folds-acoustic signal) make it challenging to isolate individual components and quantify their particular contribution to a healthy voice.^9–12^

In the clinic, many diagnostic and therapeutic procedures focus on the oscillation patterns of the vocal folds. For example, symmetry, periodicity, and full glottal closure are considered essential features of vocal fold dynamics.^13–15^ During *in vivo* examination, the quality of the resulting acoustic signal can be evaluated subjectively by auditory-perceptive measures (i.e., GRABS scale) or objectively by the computer-based calculation of acoustic parameters [i.e., harmonic-to-noise ratio (HNR), cepstral peak prominence (CPP), etc.]. In this context, a high quality of the acoustic signal is associated with a large degree of regularity, high harmonic content, and few noise components. In this paper, by “noise,” we refer to the noise in the acoustic signal generated within the phonation process and refer not to noise from electronic equipment or the surrounding environment. The term “signal quality” is equally used for the acoustic signal and the subglottal pressure signal.

Glottal closure insufficiency has generally been linked to increased broadband noise, which is thought to be one of the main factors causing hoarseness.^16,17^ However, empirical studies based on synthetic vocal folds, excised cadaver larynges, and *in vivo* measurements on healthy and disordered voices present, to some extent, contradictory findings.^18–20^

The data situation is similarly broad and inconsistent for symmetry. Asymmetric oscillation patterns were associated with irregular vibration patterns and rough voices,^4,21^ although large degrees of left-right asymmetry were found in vocally healthy subjects^22,23^ and did not necessarily diminish the acoustic signal quality in synthetic vocal folds.^24^ In the case of periodicity, systematic investigations are rare due to the limited use of high-speed cameras in diagnostics. High degrees of periodicity were reported for healthy adults and children.^25^ Periodicity of the vocal fold dynamics seems to influence the generated acoustic quality beneficially, since positive correlations were found between periodicity in vocal fold vibrations and supraglottal cepstral peak magnitude.^26^ Nevertheless, some small-scale studies showed that aperiodicity is not uncommon in healthy subjects.^27^

Phonosurgical interventions like laryngoplasty typically aim to reconstruct beneficial preconditions in the larynx, which increase phonation efficiency and enhance the quality of the acoustic output.^28–30^ To predict therapeutic success and plan an appropriate procedure, it would be highly desirable to ascertain the relative importance of the individual factors and their resulting effect.^31–33^ However, fundamental research toward this end is impeded by limited accessibility during *in vivo* examination.

A systematic analysis of excised larynges without the vocal tract offers the advantages of direct control of the laryngeal configurations and sufficient accessibility to monitor major aspects involved in generating the primary signal. The experimental design conceptualizes the larynx as a multilevel system with three distinct regions: subglottal, glottal, and supraglottal.^34^ An automated experimental setup was developed to control and measure the key aspects of phonation simultaneously across all three domains.^35,36^ The subglottal airflow is controlled while a pressure transducer monitors subglottal pressure. In contrast to the periodicity of the oscillation pattern, the symmetry and closure of the vocal folds during phonation are addressed through the pre-phonatory settings of the larynx. That is, variable laryngeal configurations are induced at the glottal level by a pre-phonatory gap in the posterior region and asymmetric adduction levels of the arytenoid cartilages. A high-speed camera registers the resulting oscillation pattern of the vocal folds, and a segmentation-based software tool enables the objective evaluation of the glottal dynamic parameters, i.e., symmetry, periodicity, and closure. A microphone in the supraglottal region records the resulting acoustic signal above the vocal folds (without vocal tract). For fundamental research on the underlying fluid-structure-acoustic interaction, *ex vivo* larynges are preferable to *in vivo* measurements. Despite the decreased comparability to *in vivo* data, the absence of a vocal tract represents a necessary compromise to enable visibility of the vocal folds in the high-speed recordings.

The multimodal measurements were analyzed with respect to clinically relevant aspects, including phonation efficiency and the acoustic signal quality quantified by established measures for noise, regularity, and harmonic content.^37–41^ Compared to earlier works, the subglottal signal was analyzed in more detail.^42–44^ Both the subglottal pressure and the supraglottal acoustic signal were recorded with the same sampling frequency and underwent identical processing steps, which enabled direct comparison and correlation of characteristic parameters (i.e., noise and perturbation measures).

Using these data, we will answer the following three research questions. (1) How do isolated glottal dynamic parameters influence the signal quality in the subglottal pressure and the acoustic signal? (2) What is the relationship between subglottal pressure and supraglottal acoustic quality? (3) What is the relative importance of the individual glottal parameters for high signal quality?

To summarize, this systematic experiment will contribute to a fundamental understanding of the signal modulation process in the directly adjacent environment of the vocal folds by investigating the interactions between the different laryngeal domains. These are inaccessible during *in vivo* recordings, and the obtained outcomes are not directly comparable to *in vivo* measurements. However, in return, it offers the chance to investigate the myoelastic-aerodynamic theory more closely. Eventually, this profound knowledge on the generation of the primary voice signal before the vocal tract will be valuable for focused therapy and precise planning of surgical interventions and behavioral treatment.

## II. METHODS

### A. Data acquisition

#### 1. Preparation of larynges

*Ex vivo* experiments enable the required accessibility to all relevant regions around the larynx during phonation. The excised porcine larynx model was chosen for its good comparability to human phonation and easier availability than human excised larynges, although porcine ventricular folds typically display the behavior of active oscillators in contrast to the ventricular folds in humans.^45,46^

**FIG. 1.**
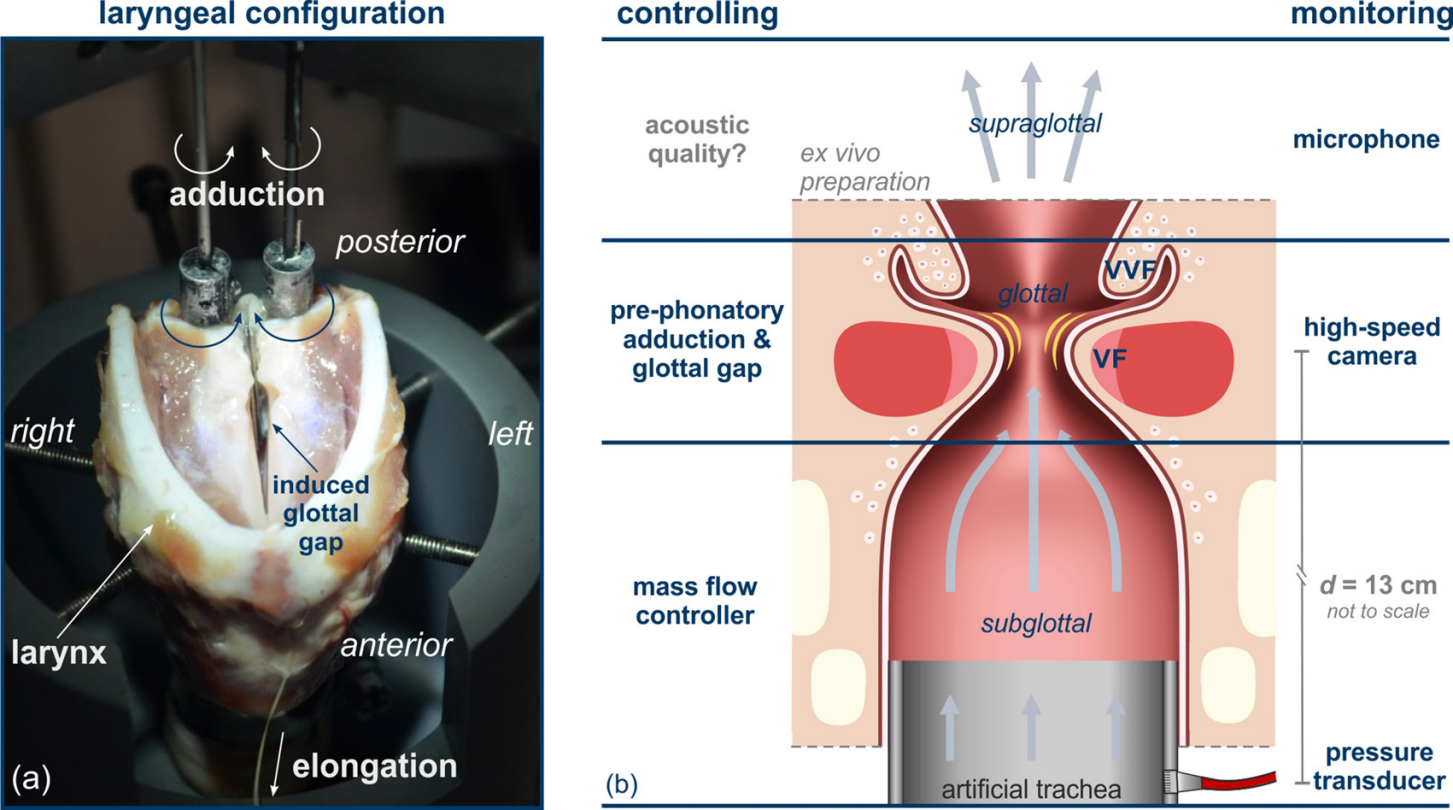
(Color online) (a) Experimental setup for manipulation of pre-phonatory configurations in excised porcine larynges. (b) Schematic overview of multilevel approach to controlling and monitoring the phonatory process in excised larynges. The distance *d* between the glottal plane and the pressure sensor is not to scale.

Nine porcine larynges (with individual identification numbers ID_larynx_ = L1–L9) were obtained from the local slaughterhouse. The larynges were quick-frozen with 2-methylbutane (–150 °C) and stored at −80 °C to preserve the tissue properties. Right before the experiments, the individual larynges were slowly thawed in a refrigerator and soaked in NaCl solution for 15 min. All supraglottal tissue was removed above the ventricular folds for an unobstructed top view on the glottal region (ventricular folds, vocal folds, and glottis) as shown in Fig. 1(a).

#### 2. Hardware: Control and measurement

The multimodal investigation of phonation in the larynx as a multilevel system is illustrated schematically in Fig. 1(b). Each of the three regions is controlled and measured separately: subglottal, glottal, and supraglottal region. Please note, “supraglottal” refers to the region immediate above the vocal folds but does not include a vocal tract in the presented experiment. Detailed information on the experimental setup can be found in Birk *et al.*^35^ The larynx was mounted on an artificial tracheal tube of stainless steel with a diameter of 20 mm. In the subglottal region, the airflow *Q* that induces the self-sustained oscillation of the vocal folds is regulated by a 1579 A/B (MKS, Andover, MA) mass flow controller and a 4000B (MKS) digital power supply. The ultrasound nebulizer Ultrasonat 810 (Hico, Hirtz & Co. KG, Köln, Germany) is interposed upstream before the artificial trachea to prevent tissue dehydration by regulating temperature and moisture to physiological levels. The temporal evolution of the pressure signal in the subglottal region, *P*_sub_, is registered 130 mm below the glottal plane through a small hole drilled in the artificial trachea. The *P*_sub_ signal is recorded for 2 s with a sampling rate of 96 kHz by an XCS-93–5PSISG (Kulite Semiconductor Products, Inc., Leonia, NJ) pressure sensor driven by a PXIe-4330 (National Instruments, Austin, TX) bridge module.

On the glottal level, a variety of oscillation patterns in the vocal folds results from different pre-phonatory laryngeal configurations. Exemplary settings are illustrated in the Appendix (see Fig. 7). Two electro-mechanic devices for posturing of the arytenoid cartilages control the vocal fold adduction. The rotating manipulation induces a torque *T_R/L_* on either side, which is measured by a TD70 (ME Meßsysteme GmbH, Hennigsdorf, Germany) sensor. A pre-phonatory gap is induced by a varying number of metal shim plates (1 mm thickness) in the posterior region of the larynx. The initial vocal fold elongation is realized by a constant weight of 50 g that is connected to the thyroid cartilage with a surgical suture. The dynamic behavior of the vocal folds and the ventricular folds is recorded by a Phantom V2511 (Vision Research, Wayne, NJ) at a frame rate of 4 kHz and a spatial resolution of 768 pixels ×768 pixels for the duration of 600 ms.

In the supraglottal region, the resulting acoustic signal is recorded for 2 s with a sampling rate of 96 kHz by a 4189 (Brüel & Kjaer, 2850 Nærum, Denmark) 1/2-in free-field microphone at 30 cm distance with an inclination of 45°. The acoustic signal was amplified by a Nexus 2690 microphone conditioning amplifier (Brüel & Kjaer) and captured by a 4492 (National Instruments) dynamic signal acquisition module.

#### 3. Measurement protocol

Resulting from numeric simulations and *in vivo* and *ex vivo* experiments, both asymmetry^3,13,47,48^ and insufficient glottal closure^19,49,50^ are strongly associated with pathologic phonation and reduced acoustic quality. In the absence of auditory-perceptive measures, i.e., GRABS scale, we tried to reflect the different aspects of acoustic quality by the use of several parameters on noise, regularity, and harmonic content (see Sec. II B 3). To generate a large variety of vocal fold oscillation patterns and to create diversity in the resulting acoustic outcome, symmetry and glottal closure were manipulated accordingly. For each larynx, three different asymmetric adduction levels *A* = *T_L_* (mNm):*T_R_* (mNm) (*A*_1_ = 5:15, *A*_2_ = 5:25, *A*_3_ = 15:25) and three different pre-phonatory gap sizes (*g*_1_ = 0 mm, *g*_2_ = 1 mm, *g*_3_ = 2 mm) were applied, which resulted in nine different laryngeal configurations. After adjusting these pre-phonatory conditions, the glottal airflow was gradually increased until sustained phonation was initiated. Starting from this phonation threshold pressure or phonation onset, the glottal flow was raised by six steps of 5 standard liters/min (SLM), resulting in a total number of seven measurements for every laryngeal configuration.

### B. Data processing and analysis

The acquired multimodal measurement signals were analyzed separately and in combination to enable an understanding of the causal relationships, coupling, and feedback effects in the fluid-structure-acoustic interaction. An overview of all computed parameters is given in Table I.

**TABLE I.** Computed parameters for aerodynamics, glottal dynamics, acoustic signal, and subglottal pressure. AU, arbitrary units.

Parameter	Abbreviation (unit)	Reference	Range/description
(a) **Aerodynamic parameters**			
Glottal flow resistance	*R_B_* (Pa/SLM)	Ref. 51	Higher is better^a^
Sound pressure level	SPL (dB)	Ref. 52	Higher is louder
(b) **Glottal dynamic parameters**			
Glottal gap index	GGI (AU)	Ref. 53	[0,1]/0: full closure
Closing quotient	CQ (AU)	Ref. 54	[0,1]/1: completely open
Amplitude periodicity	AP (AU)	Ref. 55	[0,1]/1: periodic
Time periodicity	TP (AU)	Ref. 55	[0,1]/1: periodic
Phase asymmetry index	PAI (AU)	Ref. 55	[0,1]/0: symmetric
Amplitude symmetry index	ASI (AU)	Ref. 56	[0,1]/1: symmetric
(c) **Acoustic and subglottal pressure parameters**			
Harmonics to noise ratio	HNR (dB)	Ref. 39	Higher is better^a^
Normalized noise energy	NNE (dB)	Ref. 41	Smaller is better^a^
Cepstral peak prominence	CPP (dB)	Ref. 38	Higher is better^a^
Shimmer	Shim (%)	Ref. 40	Smaller is better^a^
Jitter	Jitt (%)	Ref. 40	Smaller is better^a^

^a^ The expression “Smaller/Higher is better” refers to healthy modal phonation with respect to efficiency, regularity, harmonic content, and noise.

#### 1. Aerodynamic parameters

The glottal flow resistance *R_B_* indicates the energy transfer between the glottal flow and the vibrating tissue, thus representing a measure of phonation efficiency. It can be calculated from the average of the applied airflow $\bar{Q}$ and the measured glottal pressure difference.^51^ Without a vocal tract, as in the present setup, the pressure difference can be determined as the temporal average of the subglottal pressure signal $\bar{P_{\text{sub}}(t)}$. Furthermore, the mean sound pressure level (SPL) of each measurement is calculated from the time-resolved acoustic signal [see Table I(a)].

#### 2. Glottal dynamic parameters

The high-speed imaging recordings were analyzed with the help of a well-established software tool that was developed in-house, known as *Glottis Analysis Tools* (GAT). This software tool enables an automatic segmentation of the glottal area waveform and subsequent calculation of characteristic parameters of the vocal fold dynamics during phonation.^57^ Two representative parameters are chosen for each essential aspect of the glottal dynamics: closure [glottal gap index (GGI), closing quotient (CQ)], periodicity [amplitude periodicity (AP), time periodicity (TP)], and symmetry [phase asymmetry index (PAI), amplitude symmetry index (ASI)]. More detailed information on the glottal dynamic parameters is given in Table I(b) and in the references listed therein. To guarantee comparability of all parameters between different recordings, the analysis is performed on a signal sequence of 30 consecutive oscillation cycles in each high-speed recording. This is the highest common number of cycles in all recordings and in accordance with a suggested minimum number of 20 cycles to ensure stability in the high-speed imaging-based parameters.^58,59^

#### 3. Acoustic and subglottal pressure parameters

The time-resolved signals for acoustic and subglottal pressure were processed and evaluated in the same way.^60^ With oscillation frequencies ranging down to 50 Hz, the complete signal of 2 s length was analyzed in all recordings to reach the recommended number of at least 100 cycles.^61,62^ Both signals were filtered with a Butterworth filter in the range of 20 Hz to 20 kHz to eliminate potential background noise. The GAT software was used for automated cycle detection, determination of the fundamental frequency (*F*_0_), and evaluation of established parameters reflecting the signal quality in noise [HNR, normalized noise energy (NNE)] and regularity [jitter (Jitt), shimmer (Shim), CPP]. Further information and literature regarding these parameters are presented in Table I(c).

#### 4. Statistical analysis

From *in vivo* examinations on healthy and disordered voices, it can be expected that complete glottal closure and high degrees of periodicity and symmetry are beneficial for the resulting quality of the acoustic outcome. From the myoelastic-areodynamic theory, it can be deduced that the signal quality in the subglottal region—being the input signal to the modulation process in the larynx—will consequently influence the output signal in the supraglottal region below the vocal tract.^8^ To reveal and quantify the individual influences as well as the interrelations between the different components, the analysis of the collected parameters was structured in the following three parts. All statistics were performed using IBM SPSS version 24 (IBM, Armonk, NY). Detailed results are given in the Appendix (see Fig. 8).

***Question 1: How do isolated glottal parameters influence the quality of the acoustic signal and subglottal pressure?*** The signals were evaluated based on the CPP values of either signal, as it was shown to reliably reflect the signal quality in *ex vivo* measurements.^26,37,61^ Each glottal parameter is summarized in three cluster groups (high/medium/low) to illustrate its influence on CPP_audio_ and CPP_P__sub_. Based on the apparent behavior in the high-speed recordings, the GGI values were allocated to the following intervals: GGI_1_ ([0;0.01], entire glottal closure during vibration); GGI_2_ (]0.01;0.4[, partial glottal closure), GGI_3_ ([0.4;1], no visually recognizable contact of vocal folds). This procedure is justified by the distribution of GGI, which displays distinctly separated groups in these intervals and already provided good results in the rabbit model.^44^ Exemplary datasets for high, medium, and low quality are provided in the Appendix [see Figs. 8(a)–8(c)] with the corresponding measurements of subglottal pressure, the glottal area waveform of the high-speed recording, and the acoustic signal with the calculated values for CPP_Psub_, GGI, and CPP_audio_.

The other glottal dynamic parameters, with less obvious distributions, were subdivided objectively by *k*-means clustering into three cluster centers. The determined cluster groups did not reflect the complete possible range of each parameter but rather represented low, medium, and high intervals within the occurring measurements. In addition to graphic comparisons, a series of statistical tests was performed to investigate significant variations in signal quality for CPP_audio_ and CPP_P__sub_ due to changes in glottal parameters. A Kolmogorov–Smirnov test showed that across all investigated parameters from aerodynamics, glottal dynamics, acoustics, and subglottal pressure, only one parameter, namely SPL, was normally distributed. Nevertheless, all parameters were analyzed with non-parametric tests for mean comparison of the cluster groups. The Kruskal–Wallis test was performed with a significance level of *p* = 0.05. In significant cases, it was followed by the *post hoc* Mann–Whitney *U* test with a Bonferroni-adjusted significance level of *p* = 0.017 (= 0.05/3).

***Question 2: What is the relation between supraglottal acoustics and subglottal pressure?*** In the myoelastic-aerodynamic theory, the subglottal pressure signal represents a superposition of the initially unmodulated airflow from the lungs and the modulated components from the interaction with the vocal fold vibrations.^7,8^ It was hypothesized that the signal quality of the subglottal pressure entering the larynx strongly influences the resulting quality of the supraglottal acoustic signal above the vocal folds. For each of the acoustic and subglottal pressure parameters, the strength of this effect was investigated by the calculation of Pearson's correlation coefficients, which were determined separately for each larynx and then averaged. For each larynx, 10–63 values were considered in the correlation analysis for each parameter. Since parameter values were calculated from different signals at different measurement settings, they are considered independent from each other, being a prerequisite for Pearson's correlation.

Furthermore, a simple linear regression analysis was performed for each parameter to find the coefficients of determination *R*^2^ between the subglottal and the supraglottal parameter. Higher order regressions were not indicated, since the graphic evaluations displayed no apparent trends apart from the CPP values. In addition, the most prominent correlation between CPP_audio_ and CPP_P__sub_ was graphically evaluated regarding the beneficial influence of certain translaryngeal configurations, which boosted the resulting CPP_audio_ over-proportionately with respect to the incoming CPP_P__sub_. Due to co-linearities between the glottal dynamic parameters and CPP_P__sub_, the necessary preconditions for a multiple linear regression analysis were not met.

***Question 3: What is the relative importance of the individual glottal parameters for high signal quality?*** To ascertain the importance of the different glottal dynamic parameters, it was necessary to quantify their impact on the resulting signal quality. For this exploratory purpose, a stepwise multiple linear regression analysis was justified and performed separately on CPP_audio_ and CPP_P__sub_ as dependent variables. In addition to the glottal dynamic parameters, the *R_B_* and the identification number of each larynx ID_larynx_ (=L1–L9) were entered in the model as independent variables. Starting from an empty model, independent variables were added step by step following an automated and standardized algorithm combining forward selection (*p* < 0.05) and backward elimination (*p* > 0.1). The adjusted coefficients of determination *R^2^* in the resulting model were reported, indicating the proportion of the variance in CPP that could be explained by the independent variables. Regression coefficients and their confidence intervals were only of subordinate interest, since the prediction of CPP was not the focus of this study. The preconditions for multiple linear regression according to the Gauß–Markov theorem were considered: The independent variables were analyzed for multicollinearity, and the residuals were checked for independence, homoscedasticity, and normal distribution.

## III. RESULTS

A total of 567 datasets were acquired from nine excised larynges. Every larynx was measured with nine different glottal configurations (=3 glottal gaps × 3 asymmetric adduction levels) and seven flow steps. For 110 measurements, the larynges displayed highly erratic oscillation behavior in at least one of the signals (high-speed imaging or acoustic or subglottal pressure), preventing the determination of the *F*_0_ by means of cycle detection hindering subsequent analysis. An example of an excluded measurement with erratic oscillation characteristics and undetectable *F*_0_ in at least one of the signals is given in the Appendix [see Fig. 8(d)]. One series of 21 datasets displayed disproportionately high values of glottal flow resistance, indicating leakage in the setup. All these data were not included in the study. Hence, 436 datasets remained, being suitable for further processing, and were included in the statistical analysis.

### A. General phonation parameters

An overview of the general phonation parameters can be found in Table II, separated by the measurements of phonation onset and averaged over all datasets. The fundamental frequencies of the subglottal pressure (*F*_0__*,P*__sub_), of the glottal area waveform from the high-speed imaging (*F*_0__,__HSI_), and of the acoustic signal (*F*_0,audio_) are determined by means of cycle detection and averaged over the analyzed sequence. Minor differences between the *F*_0_ values result from the occurrence of higher harmonic oscillation modes in the three distinct regions of the larynx, which has been reported previously.^62^

**TABLE II.** Range of general phonation parameters for phonation onset and over all recordings: *F*_0_, *P*_sub_, *Q*, *R_B_*, and SPL.

	*F*_0,HSI_ (Hz)	*F*_0,audio_ (Hz)	*F*_0,__*P*__sub_ (Hz)	*P*_sub_ (Pa)	*Q* (SLM)	*R_B_* (Pa/SLM)	SPL (dB)
Phonation onset (*N* = 62)	103 ± 51	106 ± 52	100 ± 46	663 ± 258	33 ± 20	27 ± 17	82 ± 4
All recordings (*N* = 436)	109 ± 52	112 ± 54	107 ± 50	1038 ± 442	48 ± 22	26 ± 14	88 ± 5
Minimum values	48	48	48	162	8	3	75
Maximum values	280	279	279	3038	122	83	100

**FIG. 2.**
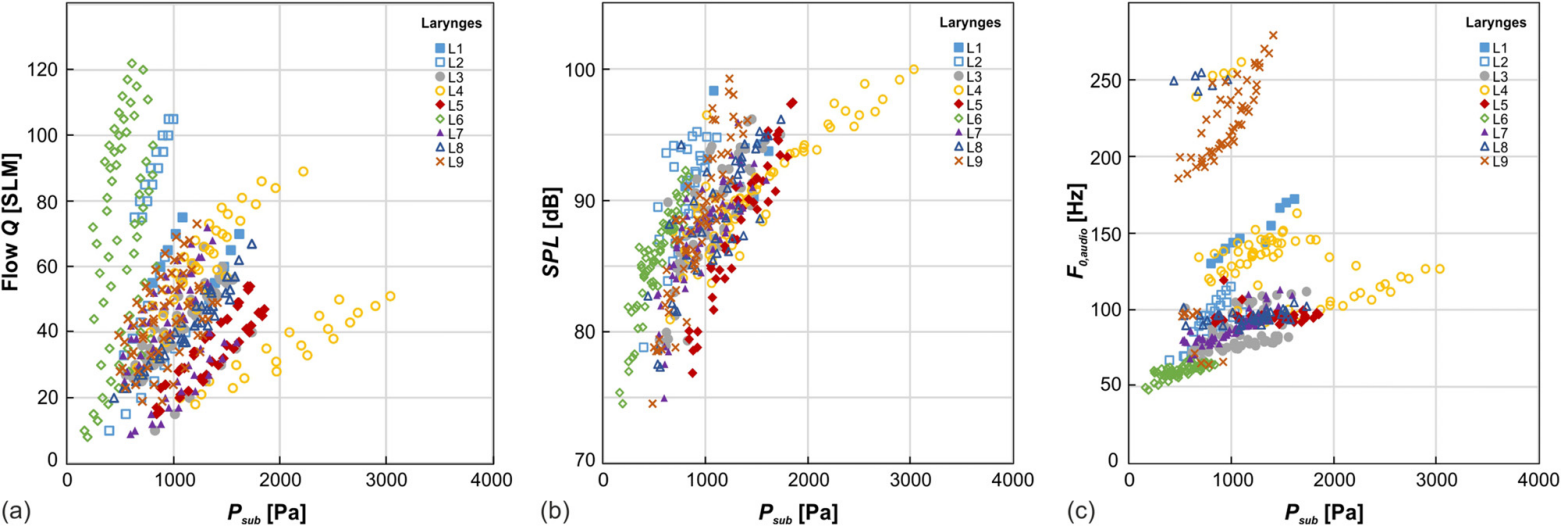
(Color online) Flow *Q*, SPL, and *F_0_*_,audio_ over *P*_sub_ for all measurements over all larynges L1–L9.

For the airflow *Q*, the SPL, and the *F_0_*_,audio_, the interindividual differences between the investigated larynges are shown in Fig. 2. In all cases, the parameters display mostly linear trends with respect to subglottal pressure *P*_sub_ and reasonable homogeneity. In Fig. 2(c), the excitation of higher oscillation modes can be observed in three of the larynges, leading to an offset from the larger point cloud.

**FIG. 3.**
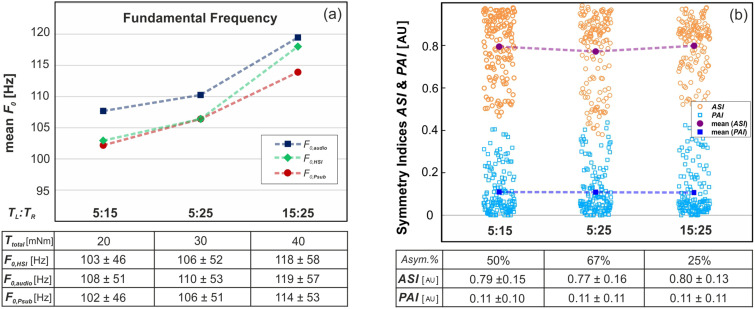
(Color online) Influence of applied adduction levels on *F*_0_ (a) and on measured asymmetry levels (b). Please note that the dashed lines are for visualization and do not indicate a linear interpolation. Below, absolute torque, asymmetry (%), and for parameters the means and standard deviation values are given.

The effect of the applied asymmetric stimulation of the arytenoid adduction on the observed oscillation patterns is depicted in Fig. 3. The determined *F*_0_ values in high-speed imaging, acoustic pressure, and subglottal pressure rise with increasing total adduction load, *T*_total_ *= T_L_* + *T_R_*. The variation between *F*_0,HSI_, *F*_0,audio_, and *F*_0__*,P*__sub_ is attributed to the occurrence of higher harmonics [see Fig. 3(a)]. Both calculated symmetry measures ASI and PAI display considerable variance and only insignificant changes in their mean values with respect to the increased level of asymmetry, *Asym.%*$=\left|T_{L}-T_{R}\right|/{(T}_{L}+T_{R})$ [see Fig. 3(b)].

### B. Statistical analysis

**FIG. 4.**
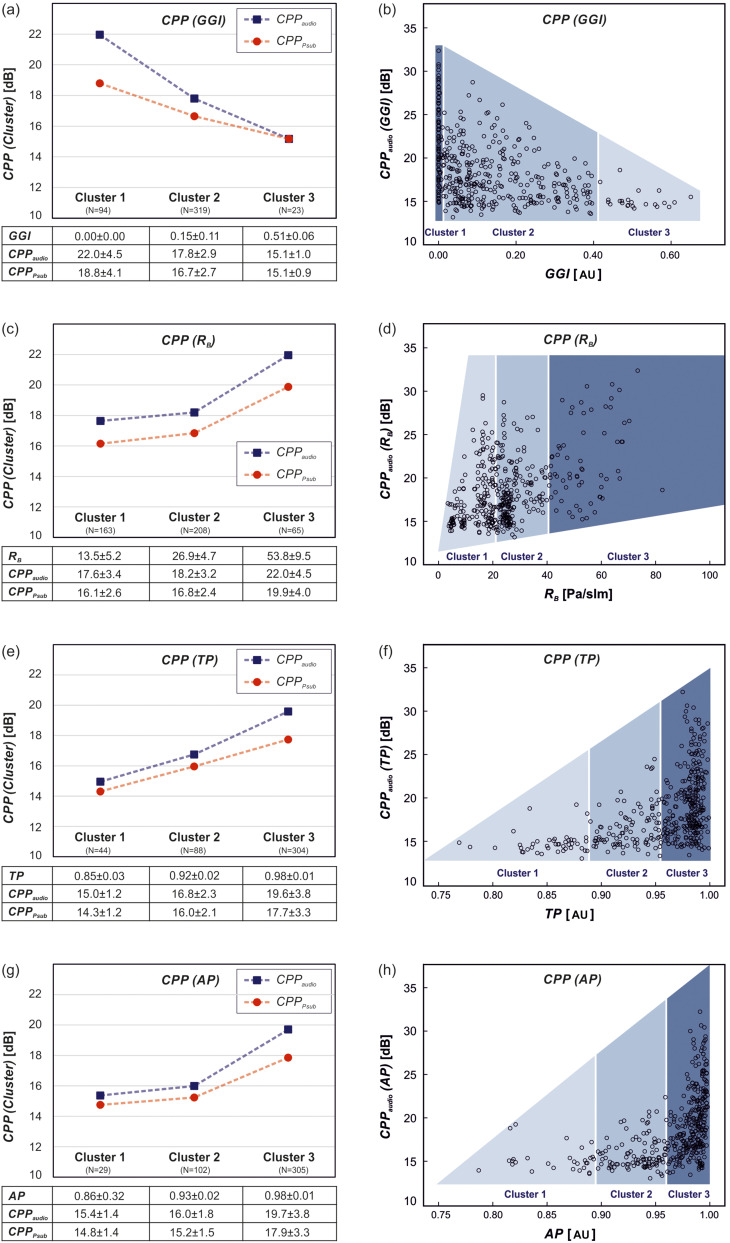
(Color online) CPP over glottal and aerodynamic parameters (GGI, RB, TP, AP). Left: Mean CPP of acoustic signal and subglottal pressure over cluster groups. Please note that the dashed lines are for visualization and do not indicate a linear interpolation. Below, means and standard deviation values are provided for each cluster. Right: Distribution of CPP of acoustic signal over parameters with assignment to their corresponding cluster group indicated by background color.

**FIG. 5.**
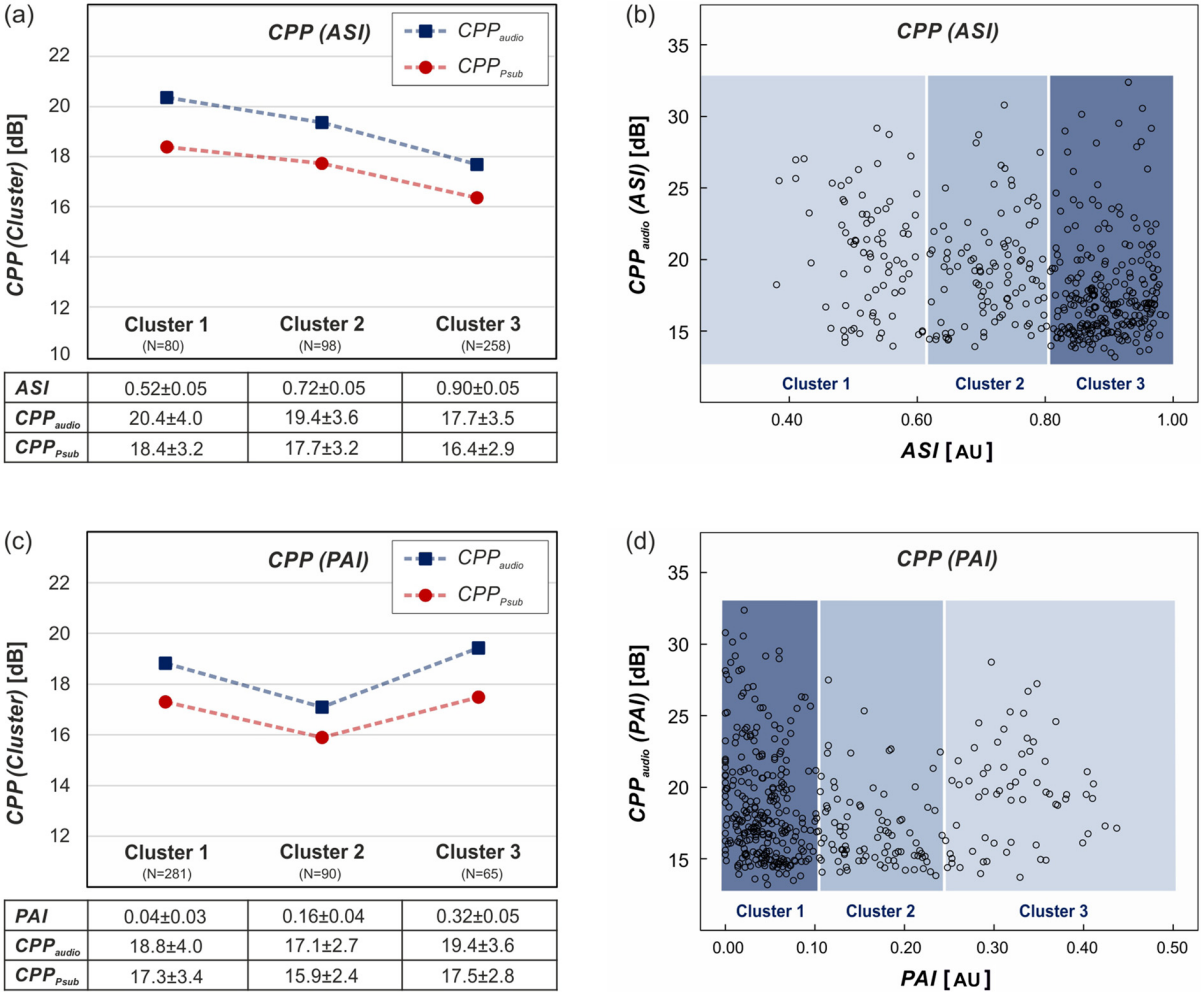
(Color online) CPP of acoustic signal and subglottal pressure over glottal parameters (ASI, PAI). Left: Mean CPP over cluster groups. Please note that the dashed lines are for visualization and do not indicate a linear interpolation. Below, means and standard deviation values are provided for each cluster. Right: Distribution of CPP of acoustic signal over parameters with assignment to their corresponding cluster group indicated by background color.

***Question 1: How do isolated glottal parameters influence the quality of acoustic signal and subglottal pressure?*** The mean values of the glottal parameters within each of the cluster groups and the corresponding mean signal quality given by CPP_audio_ and CPP_P__sub_ are presented in the left column of Figs. 4 and 5. Exact values and associated standard deviations are given in tabular form below each plot. The right column displays the exact measurements of CPP_audio_ over the continuous parameter values. Details of the statistical analysis are summarized in the Appendix (see Table V).

As can be seen in Fig. 4(a) for both signals, the CPP values decrease with increasing GGI_*i*_, which indicates an increasing glottal closure insufficiency. These changes are statistically significant in all groups. Despite strong variances, the scatterplot in Fig. 4(b) shows that larger CPP values only occur for smaller values of the GGI. It is notable that for GGI ≈ 0, the measured CPP values are quite uniformly distributed over the occurring parameter range.

Only the highest values of glottal flow resistance in *R_B/3_* generate significantly higher mean CPP values than low and medium values of *R_B_* [see Fig. 4(c)]. This is reflected in the statistical analysis yielding no significant differences between *R_B/1,2_*. Similarly, the scatterplot displays no clear linear trends in Fig. 4(d) with most data points in the lower left and large overall variance.

The two periodicity measures TP and AP display similar behavior in Figs. 4(e) and 4(g). Increased periodicity results in increased mean CPP values. For AP, only the cluster group with high periodicity differs significantly from the others, whereas all differences in TP_*i*_ proved significant (see Table V in the Appendix). In analogy to GGI, the highest values of periodicity (AP and TP close to 1) enable large CPP values but do not guarantee them [see Figs. 4(f) and 4(h)].

The results of the two symmetry measures PAI and ASI in Fig. 5 must be evaluated with caution. As shown for ASI in Fig. 5(a), the mean values of both CPP_audio_ and CPP_P__sub_ decrease with increasing degree of symmetry (ASI close to 1: symmetric), which is suggested by the statistical analysis, yielding significant differences between all cluster groups in both signals. However, the scatterplot in Fig. 5(b) displays rather uniformly distributed CPP values along the observed range of ASI with a slight accumulation of data points in the lower right corner.

The mean CPP values of both signals display no clear trend over the PAI cluster groups in Fig. 5(c). With PAI indicating symmetry close to 0, the low and high groups of PAI yield significantly higher CPP values than the medium group. Again, the scatterplot distribution reveals no distinct tendency, since high CPP values occur along the complete range of measurements [Fig. 5(d)].

***Question 2*: *What is the relation between supraglottal acoustics and subglottal pressure?*** All results of the correlation analysis between subglottal pressure and the acoustic signal are listed in Table III. Pearson's coefficients of the bivariate correlations were calculated separately for each larynx/parameter and then averaged over all larynges. All Pearson coefficients display pronounced variability among the larynges, whereas the outliers in each parameter cannot be attributed to an individual specimen but alternate between the larynges. The parameter Jitt displays the weakest correlation with highest standard deviation at *P* = 0.55 ± 0.23. By contrast, CPP shows the strongest correlation with the smallest standard deviation at *P* = 0.88 ± 0.15. The linear regression confirms a statistically significant relation (*p* < 0.001) between all parameters. Again, the CPP values yield the highest correlation and are therefore further investigated.

**TABLE III.** Correlation between subglottal pressure and supraglottal acoustic pressure signal: Bivariate correlation with Pearson's correlation coefficient *P* calculated separately for all larynges and then averaged. Linear regression is performed on all measurements for all larynges.

Audio/*P*_sub_ parameter	Bivariate correlation			Linear regression over all measurements			
	Mean *P*	Minimum *P*	Maximum *P*	*R*²	β(*P*_sub_)	β(const.)	*p*-value
HNR	0.58 ± 0.23	0.19	0.90	0.36	0.48	1.05	0.001
NNE	0.69 ± 0.19	0.35	0.85	0.30	0.42	–6.43	0.001
CPP	0.88 ± 0.15	0.47	0.99	0.67	0.98	1.94	0.001
Shim	0.68 ± 0.18	0.32	0.91	0.56	0.76	4.01	0.001
Jitt	0.55 ± 0.23	0.15	0.90	0.42	0.56	2.61	0.001

**FIG. 6.**
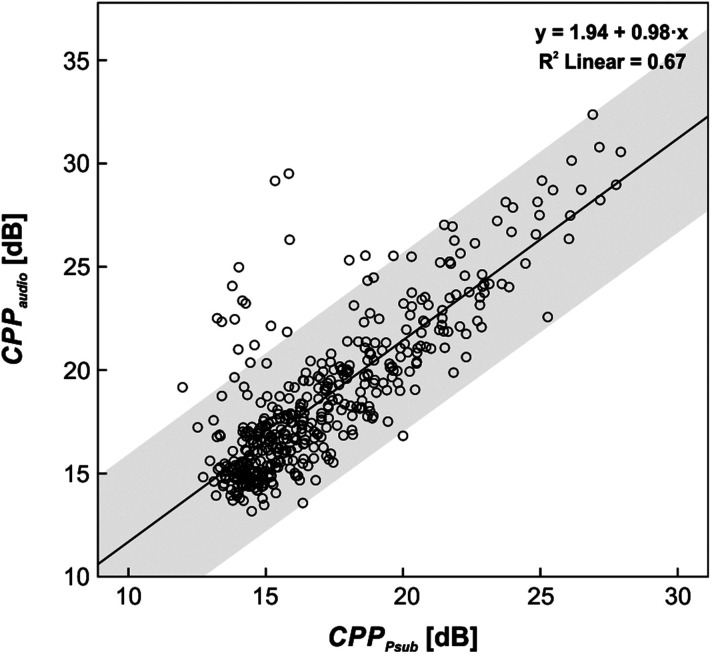
CPP_audio_ of acoustic signal over CPP_P__sub_ of subglottal pressure with linear regression line, corresponding regression coefficients (upper corner), and 95% confidence interval (gray).

**FIG. 7.**
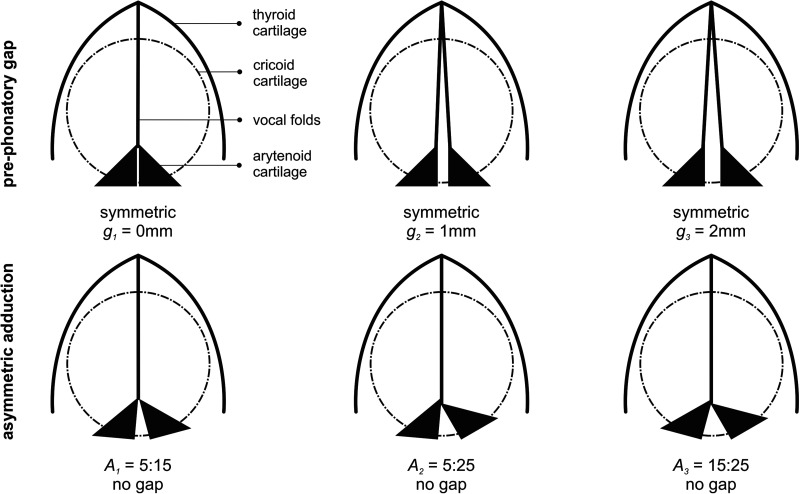
Exemplary laryngeal configurations: different sizes of pre-phonatory gap (upper row) and different degrees of asymmetric adduction (lower row).

**FIG. 8.**
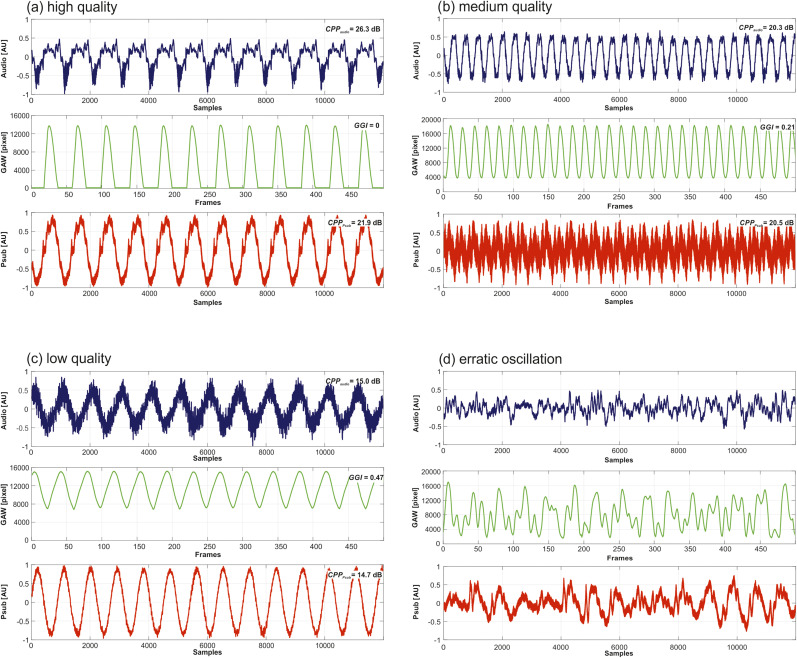
(Color online) Exemplary datasets for (a) high, (b) medium, and (c) low quality with the corresponding measurements of the subglottal pressure, the glottal area waveform (GAW) of the high-speed recording, and the acoustic signal with the calculated values for CPP_P__sub_, GGI, and CPP_audio_ and (d) an excluded measurement displaying erratic behavior (undetectable *F*_0_) in at least one signal. In all cases, the length of the depicted signal sequence is 125 ms, which corresponds to 500 frames in the high-speed recordings (4 kHz) and 12 000 samples in subglottal pressure and acoustic signal (96 kHz).

The general linear trend between CPP_audio_ and CPP_Psub_ over all measurements is shown in Fig. 6 with a linear fitting function. A linear regression showed correlation (*R*^2^ = 0.67; F(1,434) = 865.51; *p* < 0.001) and provided regression coefficients *β*(CPP_P__sub_) = 0.98 ± 0.03 and *β*(const.) = 1.94 ± 0.57. This is in accordance with the predominantly parallel behavior of the averaged CPP values in the left column of Figs. 4 and 5. Over all glottal parameters and all cluster groups, the mean values of CPP_audio_ are generally larger than CPP_P__sub_ (or equal in the case of GGI_3_), reflecting an increase in signal quality in flow direction and upward through the larynx, respectively. In all cases, the acoustic signal covers a larger range of CPP values compared to subglottal pressure. However, despite the strong linear correlation between subglottal pressure and acoustic signal, we observe an over-proportional improvement of CPP_audio_ for increasing closure (i.e., with decreasing GGI) [Fig. 4(a)]. It is noteworthy that for GGI_3_, no contact of the vocal folds, the signal quality persists unchanged from the subglottal to the supraglottal region. Likewise, for the periodicity measures TP [Fig. 4(e)] and AP [Fig. 4(g)], the boosting effect can be observed, but only for the highest cluster group TP_3_ and AP_3_, while the others behave in a parallel manner. As for the other glottal dynamic parameters *R_B_*, ASI, and PAI, the mean CPPs remain mostly parallel over the complete parameter range.

The other noise and perturbation measures for the acoustic signal and subglottal pressure do not show a consistent pattern. For example, the mean values of all phonation parameters were calculated against the influence of three glottal characteristics: symmetry, closure, and periodicity. For the sake of brevity, only one parameter was chosen for each characteristic: ASI, GGI, and TP. The mean values and statistical analysis over the cluster groups are given in the Appendix: ASI in Table VI, GGI in Table VII, and TP in Table VIII. It can be seen that clustered mean values for HNR_*P*__sub_ are generally higher than for HNR_audio_ and that mean values of NNE_*P*__sub_ are smaller than NNE_audio_. Both trends indicate less noise and therefore higher signal quality in the subglottal pressure signal. The CPP values are consistently higher for the acoustic signal, representing higher harmonic content of the signal in the supraglottal region. The mean Shim indicates higher amplitude fluctuations in the supraglottal region by constantly larger values in the acoustic signal. For mean Jitt values, there is no clear trend comparing the acoustic signal to subglottal pressure.

***Question 3: What is the relative importance of the individual glottal parameters for high signal quality?*** A stepwise linear regression is performed on CPP_audio_ as dependent variable with the independent variables RB, GGI, CQ, TP, AP, ASI, and PAI and the identification number of each larynx ID_larynx_. The adjusted *R*^2^ values show that 51% of the variance in CPP_audio_ can be explained by four glottal parameters as shown in Table IV. The differences (Δ*R*^2^) between the *R*^2^ values indicate the contribution of each additional independent variable. It is found that the main impact results from TP and GGI with (Δ*R*^2^ ≥ 20%). Symmetry measures (PAI, ASI), the duration of the closed phase (CQ), or interindividual differences considered by ID_larynx_ do not make a substantial contribution (Δ*R*^2^ < 0.02). In the case of CPP_P__sub_, 39% of the variance can be explained by only two variables, *R_B_* and AP. All other parameters on closure, symmetry, and the larynx itself display no considerable impact (Δ*R*^2^ < 0.02).

**TABLE IV.** Results of stepwise linear regression on CPP_audio_ and CPP_P__sub_ as dependent variables with independent variables included up to Δ*R*^2^ ≥ 0.02. Adjusted *R*^2^ penalizes for an increasing number of included independent variables.

Dependent variable	Included independent variable	Adjusted *R²*	Δ*R*²	*F*-statistic	*p*-value
CPP_audio_	TP	0.22	0.22	F(1,434) = 126.9	0.001
	TP, GGI	0.42	0.20	F(2,433) = 159.0	0.001
	TP, GGI, *R_B_*	0.46	0.04	F(3,432) = 126.2	0.001
	TP, GGI, *R_B_*, AP	0.51	0.04	F(4,431) = 112.3	0.001
CPP_P__sub_	*R_B_*	0.22	0.22	F(1,434) = 121.8	0.001
	*R_B_*, AP	0.39	0.17	F(2,433) = 139.1	0.001

In both cases, the collinearities among the independent variables are uncritical (Pearson *P* ≤ 0.65). The independence, homoscedasticity, and normal distribution of the residuals were evaluated graphically and showed acceptable levels.

## IV. DISCUSSION

### A. General phonation parameters

The general phonation parameters, as shown in Table II and Fig. 2, are consistent with previous *ex vivo* experiments of excised porcine larynges using the automated setup.^37^ However, the parameter values cover a wider range than Alipour and Jaiswal reported without the induction of a pre-phonatory gap and new adduction procedure.^45,46^ The relationship between airflow *Q* and *P*_sub_ displays a predominantly linear behavior over all larynges [see Fig. 2(a)], which is consistent with previous work,^42,62^ even though non-linear relationships have been reported as well.^46^ The SPL in Fig. 2(b) exhibits a logarithmic trend with respect to the mean subglottal pressure, as described by Björklund and Sundberg.^52^ All aerodynamic parameters are reasonably homogeneous among the measured larynges, giving no indication of systematic errors.

The automated experimental setup generates a large variety of asymmetric oscillation patterns. However, the measured symmetry parameters PAI and ASI do not show a statistically significant correlation with the applied asymmetric adduction levels (Fig. 3), which suggests that the positioning of the arytenoid cartilages is not the primary source of oscillation symmetry. Possible alternatives like a slight imbalance of the internal morphological structure of the vocal folds have to be further investigated.^63^ Nevertheless, the induced torques have an undeniable elongation effect on the vocal folds, resulting in an increase in the *F*_0_ with increasing total adduction load *T*_total_ = *T_L_* + *T_R_*.

### B. Influence of laryngeal configurations on signal quality

A pronounced beneficial influence of increased glottal closure on the CPP_P__sub_ and CPP_audio_ can be observed, which is in accordance with previous findings.^42,44^ A more detailed illustration, like the scatterplot, reveals that full closure at a GGI ≈ 0 indeed enables the complete range of signal quality but does not guarantee the highest quality results, as might be concluded from the averaged plots. However, higher CPP values become more unlikely for increasingly insufficient closure.

A similar behavior can be observed for the periodicity measures TP and AP, where the highest values of CPP only occur for highly periodic oscillations (cluster 3). However, even in this cluster 3, the complete range of CPP values is represented, see Fig. 4(f). This is in line with Mehta *et al.*, who found a positive correlation between periodicity and CPP_audio_ in patients with vocal fold lesions undergoing microsurgery.^26^ Most *in vivo* studies on healthy subjects report very high periodicity values close to 1,^53,64^ whereas other studies found aperiodic oscillations in up to 30% of healthy female subjects.^41^ Further systematic *in vivo* studies on healthy subjects and different pathologies are needed in this area.

The glottal flow resistance, which is more associated with phonation efficiency than with signal quality, also has a beneficial influence on the CPP values, however, less pronounced and with large variances in the scatterplot. In part, this may be attributed to collinearities between glottal closure (GGI) and *R_B_*, which seems to be consistent throughout most *ex vivo* mammal models.^42,44,63,65^

Deceptively, the averaged symmetry measures ASI and PAI displayed a seemingly negative influence on the signal quality. However, the scatter plots show no clear influence or causal relation, which is further endorsed by the statistical analysis on mean values in the Appendix. This contradicts previous studies on porcine larynges indicating a positive correlation between symmetry and CPP_audio_.^42^ Furthermore, *in vivo* studies found increased left-right asymmetry in patients with various laryngeal disorders,^48^ and numeric simulations displayed a decrease in voice quality with increased asymmetry in the vocal fold vibrations.^47^ Further research on the relevance of symmetric oscillations for high acoustic quality is necessary.

### C. Relation between subglottal pressure and acoustic signal

The statistical analysis confirmed a strong correlation between the noise and perturbation parameters on the acoustic signal and subglottal pressure. It can be excluded that the considerable variability in the strength of the correlation coefficients results from systematic errors or aberrations of individual larynges. With the highest sensitivity to glottal influences and greatest correlation strength, the parameters CPP_audio/__*P*__sub_ proved to be ideal for further investigations. The steeper rise of the CPP_audio_ values compared to CPP_P__sub_ indicates a stronger influence of all glottal parameters toward the supraglottal direction [see Figs. 4(a), 4(e), and 4(g)]. Evidently, the subglottal level is also influenced by the laryngeal configurations. However, the effects are superimposed, since the modulated airflow from the glottal plane mixes with the incoming unmodulated airflow from below. Despite the overall linear relation with *β*(CPP_P__sub_) ≈ 1, an over-proportional improvement is observed for beneficial laryngeal configurations. The mean CPP values display continuous divergence over the GGI_*i*_ cluster groups and for the highly periodic cluster groups TP_3_ and AP_3_. Increased *R_B_* values are generally beneficial for both CPPs, but the parallel (not divergent) behavior of the two CPPs indicates no improvement through the glottal plane.

In comparison to CPP, correlations between subglottal and supraglottal signal were less pronounced for the other parameters HNR, NNE, Shim, and Jitt. The noise measures (HNR, NNE) and the amplitude fluctuation (Shim) indicated better signal quality in the subglottal region despite the superposition of the modulated and unmodulated signals. The deterioration of these parameters in the supraglottal region can be attributed to larger aerodynamic turbulences and their resulting acoustics above the vocal folds. The period perturbation measure Jitt displayed no clear tendency regarding sub- and supraglottal signals.

It might seem contradictory that the CPP resulted in higher quality in the supraglottal region. However, the parameter CPP reacts to a wider range of signal features (i.e., fluctuations of frequency and amplitude, HNR, and the number of higher harmonics). Even though the characteristics of the other parameters are implied in the CPP to some degree, the CPP with its focus on the spectral domain behaves oppositely to the parameters describing its different aspects individually. However, this is *per se* not a contradiction but rather a motivation to perform further theoretical and experimental research on this.

### D. Quantifying relevance of glottal parameters for acoustic outcome

A quantification of relevant influences on the phonatory process, as reliable predictors for signal quality, is inherently challenging, since a fluid-structure-acoustic interaction naturally shows multicollinearity, which inhibits statistical analysis. However, the stepwise multiple linear regression can provide reasonably firm indications of statistically significant parameters in the phonation process. The results imply that TP and GGI are most important for the quality of the acoustic outcome (CPP), whereas spatial and temporal symmetry have no discernible influence. In favor of the excised larynx model, it can be stated that interindividual differences between the specimens do not play a role. The unaccounted percentage of 49% in the stepwise multiple linear regression for CPP_audio_, which the statistical model cannot explain by glottal dynamic parameters, is mainly attributed to the initial quality of the incoming subglottal signal (CPP_P__sub_). Then again, the subglottal signal is also influenced by glottal parameters, primarily by *R_B_* and AP.

The contribution of *R_B_* is a rather obvious factor, since it is calculated from the mean subglottal pressure.^51^ An increased correlation between AP and aerodynamic factors, such as airflow, lung volume, and pressure, has already been mentioned by Hirano *et al.*^66^ However, further research on the underlying causality is still pending. It is noteworthy that the two primary contributing factors to each signal are different: i.e., TP and GGI for CPP_audio_ and *R_B_* and AP for CPP_P__sub_.

To compensate for the increased chance of type I errors in stepwise multiple regression, as reported by Mundry *et al.*,^67^ we confirmed our findings by recalculation of the model while simultaneously entering the independent variables leading to the same significances. Considering the intrinsic collinearities, the stepwise multiple linear regression analysis cannot accomplish an exact prediction of the signal quality by means of the derived *β*-coefficients. However, it provides a grading of influence for the individual glottal characteristics: (1) any increase in glottal closure leads to an increase in the quality of the supraglottal acoustic outcome, (2) highly periodic vocal fold oscillations tend to yield higher supraglottal acoustic quality, and (3) the impact of asymmetry on the generated signal quality seems negligible.

These indications from multiple linear regression are supported by clustered mean values and their statistical comparisons in the Appendix.

The mean values with respect to the clustered symmetry groups ASI_*i*_ in Table VI display no significant effects on the efficiency of phonation (SPL, *R_B_*) or on the parameters of the acoustic signal and subglottal pressure. The other glottal parameters on closure, periodicity, and even the other symmetry index PAI show only minor variation among the cluster groups and/or no statistical significance between the groups. Moreover, none of the acoustic parameters is statistically significant throughout all three cluster groups. All in all, this confirms the impression of the generally minor role of symmetry in the phonation process.

The mean values over the cluster groups of GGI_i_ in Table VII reflect a distinct increase in efficiency in *R_B_* and SPL for increased closure. Beyond that, the parameters on the acoustic signal and the subglottal pressure tend toward higher signal quality, especially in CPP but not in Jitt and Shim. Apparently, the other glottal parameters are barely influenced by the glottal closure, as they display only slight variability and no consistent general trends. All in all, the glottal closure has an undeniable positive influence on the signal quality in CPP, but even more so on the phonation efficiency and sound pressure level.

The mean values for the TP_*i*_ periodicity clusters in Table VIII demonstrate a pronounced and statistically significant positive effect on all parameters of the subglottal pressure and acoustic signal. Additionally, the phonation efficiency indicated by the aerodynamic parameters is increased for higher values of periodicity. It is noteworthy that the asymmetry measures and GGI display no significant collinearity with TP. As a consequence, a confounding effect can be excluded, where TP actually favors the GGI and only as a result the signal quality. The comparative analysis indicates that periodic vocal fold oscillations and glottal closure are similarly important for the resulting quality (CPP) of the acoustic signal, whereas glottal closure primarily benefits the efficiency (*R_B_*) and intensity of phonation (SPL).

### E. Limitations

Unfortunately, fluid-structure-acoustic interactions have so far been rarely investigated under realistic conditions due to difficulties in *in vivo* accessibility. The direct measurement of the subglottal pressure and the glottal resistance during an *in vivo* examination requires tracheal puncture and is therefore extremely rare.^68^ Indirect measurements with oral pressure techniques only enable an estimation of the actual conditions in the area close to the larynx.^69,70^ This is why no meaningful quantitative comparisons to our findings can be drawn at this stage.

The presented measurements on porcine cadaver larynges exhibit the commonly known limitations of *ex vivo* experiments: (1) mechanical cartilage manipulation in contrast to physiological nerve stimulation; (2) removal of the vocal tract for an unobstructed view of the high-speed camera on the oscillating vocal folds; (3) porcine instead of human larynges. Naturally, this limits the one-to-one comparability of the quantitative parameter values to acoustic measurements from clinical data.

The findings of our experiments are certainly limited in the direct transferability and practical applicability to clinical routine but represent a valuable contribution to a deeper understanding of underlying principles in the phonatory process.

## V. CONCLUSION

To investigate the fluid-structure-acoustic interaction during the phonation process, a multimodal approach was used to control and simultaneously measure the key aspects of the primary signal generation. An excised porcine larynx model provided access to the crucial regions of interest: subglottal, glottal, and supraglottal level. A systematic variation of the oscillation patterns is effected by means of the subglottal airflow and the laryngeal configurations, i.e., a pre-phonatory gap and asymmetric adduction were induced. The glottal dynamic parameters reflecting the three allegedly essential characteristics of vocal fold oscillations (closure, symmetry, and periodicity) were calculated from high-speed recordings. Statistical methods revealed the influence of glottal dynamic parameters on typical noise and perturbation measures evaluating the signal quality of the subglottal pressure and the acoustic signal.

This study strongly suggests that symmetry is negligible for the general outcome, whereas both GGI and TP represent important contributing factors to high efficiency and signal quality in the phonation process. Increased glottal closure is especially beneficial for *R_B_* as an indicator for phonation efficiency, but also for the signal quality (CPP) in subglottal pressure and the acoustic signal. Conversely, periodicity displays a distinct positive and statistically significant influence on all noise and perturbation measures of subglottal pressure and the acoustics (including CPP) while enhancing the phonation efficiency only to a smaller degree.

The equivalent analysis of the time-resolved pressure signals in the subglottal and supraglottal region of the larynx revealed valuable insights into the modulation of the airflow throughout the larynx. Some of the investigated glottal dynamic parameters had a significant impact not only in flow direction, but in both directions on the subglottal pressure and on the supraglottal acoustic signal. Additionally, a strong linear correlation between CPP_P__sub_ and CPP_audio_ was found (*y* ≈ 0.98·*x* + 1.94). On the one hand, the constant offset, which indicates a lower quality (lower harmonic content) in the pressure signal in the subglottal region, can be explained by superposition of the modulated (reverting) airflow from the glottal region with the unmodulated airflow from below. On the other hand, the quality in the supraglottal region appears to experience a boost of quality with increasing closure, while it remains unchanged for the measurements with no vocal fold contact. The same over-proportional increase in quality between subglottal and supraglottal region can be observed for high degrees of periodicity.

The described self-enhancing effect on signal quality is analogous to the manifestation of resonances in the supraglottal vocal tract. In conclusion, it can be stated that the subglottal region and its corresponding pressure signal have an undeniable influence on the outcome of the primary acoustic signal directly above the vocal folds, which is so far scarcely investigated due to its difficult *in vivo* accessibility. Further fundamental research with *ex vivo* experiments and numeric simulations will be indispensable for a comprehensive understanding of the phonatory process and for deducing therapeutic implications for the clinic.

## APPENDIX

### 1. Schematic representation of manipulation of the larynx in the glottal region

See Fig. 7 for different pre-phonatory laryngeal configurations.

### 2. Exemplary oscillation characteristics

See Fig. 8 for multimodal datasets of high, medium, and low quality as well as erratic oscillation behavior.

#### Statistical analysis

See Tables V–VIII for detailed results of statistical tests.

**TABLE V.** *p*-values (Kruskal–Wallis test and Mann–Whitney *U* test) for comparison of CPP_audio_ and CPP_P__sub_ between cluster groups of parameters. Significant *p*-values are highlighted in bold type.

Parameter	Kruskal–Wallis (***p*** **<** **0.05**)	*Post hoc* test: Mann–Whitney *U* (***p* < 0.017**)		
CPP_audio_	GGI_1–3_	GGI_1,2_	GGI_1,3_	GGI_2,3_
	**0.000**	**0.000**	**0.000**	**0.000**
	*R_B_*_/1–3_	*R_B_*_/1,2_	*R_B_*_/1,3_	*R_B_*_/2,3_
	**0.000**	0.017	**0.000**	**0.000**
	TP_1–3_	TP_1,2_	TP_1,3_	TP_2,3_
	**0.000**	**0.000**	**0.000**	**0.000**
	AP_1–3_	AP_1,2_	AP_1,3_	AP_2,3_
	**0.000**	0.088	**0.000**	**0.000**
	ASI_1–3_	ASI_1,2_	ASI_1,3_	ASI_2,3_
	**0.000**	**0.000**	**0.000**	0.065
	PAI_1–3_	PAI_1,2_	PAI_1,3_	PAI_2,3_
	**0.000**	**0.000**	0.104	**0.000**
CPP_P__sub_	GGI_1–3_	GGI_1,2_	GGI_1,3_	GGI_2,3_
	**0.000**	**0.000**	**0.000**	0.026
	*R_B_*_/1–3_	*R_B_*_/1,2_	*R_B_*_/1,3_	*R_B_*_/2,3_
	**0.000**	**0.001**	**0.000**	**0.000**
	TP_1–3_	TP_1,2_	TP_1,3_	TP_2,3_
	**0.000**	**0.000**	**0.000**	**0.000**
	AP_1–3_	AP_1,2_	AP_1,3_	AP_2,3_
	**0.000**	0.022	**0.000**	**0.000**
	ASI_1–3_	ASI_1,2_	ASI_1,3_	ASI_2,3_
	**0.000**	**0.000**	**0.000**	0.105
	PAI_1–3_	PAI_1,2_	PAI_1,3_	PAI_2,3_
	**0.000**	**0.000**	0.300	**0.000**

**TABLE VI.** Statistical analysis of the influence of the ASI. Left: Mean values and standard deviations calculated for cluster groups of ASI. Right: *p*-values (Kruskal–Wallis and Mann–Whitney–U) for comparison between cluster groups of ASI for phonation parameters (aerodynamic, glottal, acoustic, and subglottal pressure signal). Significant *p*-values are highlighted in bold type.

Parameter	ASI_1_	ASI_2_	ASI_3_	Kruskal–Wallis (***p* < 0.05**)	*Post hoc* test: Mann–Whitney U (***p* < 0.017**)		
ASI	0.90 ± 0.88	0.72 ± 0.05	0.52 ± 0.05	ASI_1–3_	ASI_1,2_	ASI_1,3_	ASI_2,3_
Aerodynamic parameters				Aerodynamic parameters			
*P*_sub_	1040 ± 469	1094 ± 441	964 ± 333	0.211	**—**	**—**	**—**
*R_B_*	24.2 ± 13.5	30.1 ± 16.0	25.1 ± 13.9	**0.000**	**0.000**	0.820	0.084
SPL	88.4 ± 4.9	88.1 ± 4.9	88.4 ± 4.5	0.564	**—**	**—**	**—**
Glottal dynamic parameters				Glottal dynamic parameters			
PAI	0.10 ± 0.09	0.12 ± 0.13	0.13 ± 0.13	0.884	**—**	**—**	**—**
GGI	0.14 ± 0.14	0.12 ± 0.15	0.13 ± 0.15	0.183	**—**	**—**	**—**
CQ	0.44 ± 0.13	0.40 ± 0.10	0.40 ± 0.13	**0.001**	**0.000**	**0.020**	**0.977**
AP	0.96 ± 0.04	0.97 ± 0.03	0.98 ± 0.03	**0.000**	**0.003**	**0.000**	**0.000**
TP	0.95 ± 0.05	0.96 ± 0.03	0.97 ± 0.05	**0.000**	**0.004**	**0.000**	**0.001**
Acoustic parameters				Acoustic parameters			
HNR_audio_	4.7 ± 5.7	5.9 ± 4.6	7.1 ± 4.8	**0.021**	0.283	**0.008**	0.079
NNE_audio_	–12.7 ± 5.2	–13.7 ± 4.5	–14.2 ± 4.5	**0.050**	**—**	**—**	**—**
CPP_audio_	17.7 ± 3.5	19.4 ± 3.6	20.3 ± 4.0	**0.000**	**0.000**	**0.000**	0.065
Shim%_audio_	8.7 ± 6.0	6.9 ± 4.0	6.2 ± 4.5	**0.001**	0.110	**0.001**	0.026
Jitt%_audio_	7.2 ± 5.6	5.3 ± 4.1	4.7 ± 4.8	**0.000**	**0.014**	**0.000**	0.059
Subglottal pressure parameters				Subglottal pressure parameters			
HNR_*P*__sub_	8.3 ± 7.1	9.2 ± 5.8	13.5 ± 5.7	**0.000**	0.146	**0.000**	**0.000**
NNE_*P*__sub_	–14.7 ± 6.8	–16.7 ± 5.1	–19.2 ± 4.7	**0.000**	**0.008**	**0.000**	**0.000**
CPP_P__sub_	16.4 ± 2.9	17.7 ± 3.2	18.4 ± 3.2	**0.000**	**0.000**	**0.000**	0.105
Shim%_*P*__sub_	6.2 ± 5.9	4.0 ± 4.0	2.8 ± 3.8	**0.000**	**0.006**	**0.000**	**0.000**
Jitt%_*P*__sub_	7.4 ± 6.3	6.9 ± 6.2	4.3 ± 4.9	**0.000**	0.841	**0.000**	**0.000**

**TABLE VII.** Statistical analysis of the influence of GGI. Left: Mean values and standard deviations calculated for cluster groups of GGI. Right: *p*-values (Kruskal–Wallis and Mann–Whitney–*U*) for comparison between cluster groups of GGI for phonation parameters (aerodynamic, glottal, acoustic, and subglottal pressure signal). Significant *p*-values are highlighted in bold type.

Parameter	GGI_1_	GGI_2_	GGI_3_	Kruskal–Wallis (***p* < 0.05**)	*Post hoc* test: Mann–Whitney *U* (***p* < 0.017**)		
GGI	0.00 ± 0.00	0.15 ± 0.11	0.51 ± 0.06	GGI_1–3_	GGI_1,2_	GGI_1,3_	GGI_2,3_
Aerodynamic parameters				Aerodynamic parameters			
*P*_sub_	1209 ± 651	1010 ± 354	729 ± 156	**0.000**	**0.023**	**0.000**	**0.000**
*R_B_*	38.5 ± 18.7	23.3 ± 10.6	11.5 ± 3.3	**0.000**	**0.000**	**0.000**	**0.000**
SPL	89.5 ± 5.8	88.2 ± 4.4	85.5 ± 5.7	**0.005**	0.024	**0.005**	0.031
Glottal dynamic parameters				Glottal dynamic parameters			
PAI	0.04 ± 0.05	0.13 ± 0.11	0.08 ± 0.06	**0.000**	**0.000**	**0.000**	0.147
ASI	0.76 ± 0.17	0.80 ± 0.14	0.69 ± 0.18	**0.003**	**0.023**	0.156	**0.005**
CQ	0.32 ± 0.14	0.46 ± 0.11	0.46 ± 0.09	**0.000**	**0.000**	**0.000**	0.529
AP	0.97 ± 0.03	0.96 ± 0.04	0.98 ± 0.01	**0.000**	**0.003**	0.298	**0.001**
TP	0.97 ± 0.03	0.95 ± 0.05	0.99 ± 0.01	**0.000**	**0.001**	**0.025**	**0.000**
Acoustic parameters				Acoustic parameters			
HNR_audio_	6.9 ± 4.5	5.2 ± 5.8	4.7 ± 1.8	**0.018**	0.032	**0.007**	**0.138**
NNE_audio_	–13.8 ± 4.7	–13.0 ± 5.1	–13.2 ± 2.7	0.319	**—**	**—**	**—**
CPP_audio_	22.0 ± 4.5	17.8 ± 2.9	15.1 ± 1.0	**0.000**	**0.000**	**0.000**	**0.000**
Shim%_audio_	6.1 ± 4.1	8.5 ± 5.8	6.2 ± 1.9	**0.000**	**0.000**	**0.020**	0.589
Jitt%_audio_	3.8 ± 3.7	6.8 ± 5.5	9.7 ± 2.9	**0.000**	**0.000**	**0.000**	**0.001**
Subglottal pressure parameters				Subglottal pressure parameters			
HNR_*P*__sub_	11.2 ± 7.4	8.8 ± 6.8	11.4 ± 3.6	**0.009**	**0.006**	0.970	**0.078**
NNE_*P*__sub_	–16.1 ± 6.4	–15.7 ± 6.4	–19.8 ± 4.6	**0.007**	0.592	**0.008**	**0.002**
CPP_P__sub_	18.8 ± 4.1	16.7 ± 2.7	15.2 ± 0.9	**0.000**	**0.000**	**0.000**	**0.026**
Shim%_*P*__sub_	4.0 ± 4.7	5.6 ± 5.6	2.3 ± 0.9	**0.000**	**0.000**	0.424	0.024
Jitt%_*P*__sub_	4.5 ± 5.9	7.4 ± 6.2	5.4 ± 2.2	**0.000**	**0.000**	**0.001**	0.747

**TABLE VIII.** Statistical analysis of the influence of TP. Left: Mean values and standard deviations calculated for cluster groups of TP. Right: *p*-values (Kruskal–Wallis and Mann–Whitney *U*) for comparison between cluster groups of TP for phonation parameters (aerodynamic, glottal, acoustic, and subglottal pressure signal). Significant *p*-values are highlighted in bold type.

Parameter	TP_1_	TP_2_	TP_3_	Kruskal–Wallis (***p* < 0.05**)	*Post hoc* test: Mann–Whitney *U* (***p* < 0.017**)		
TP	0.85 ± 0.03	0.92 ± 0.02	0.98 ± 0.01	TP_1–3_	TP_1,2_	TP_1,3_	TP_2,3_
Aerodynamic parameters				Aerodynamic parameters			
*P*_sub_	965 ± 529	1245 ± 440	989 ± 411	**0.000**	**0.016**	0.481	**0.000**
*R_B_*	16.2 ± 12.4	28.9 ± 11.6	26.5 ± 14.9	**0.000**	**0.000**	**0.000**	**0.005**
SPL	87.7 ± 3.8	90.0 ± 4.8	88.0 ± 4.9	**0.001**	**0.003**	0.533	**0.001**
Glottal dynamic parameters				Glottal dynamic parameters			
GGI	0.12 ± 0.09	0.13 ± 0.12	0.14 ± 0.15	0.822	**—**	**—**	**—**
CQ	0.52 ± 0.09	0.43 ± 0.10	0.41 ± 0.13	**0.000**	**0.000**	**0.000**	0.162
PAI	0.11 ± 0.09	0.11 ± 0.11	0.11 ± 0.11	0.424	**—**	**—**	**—**
ASI	0.82 ± 0.10	0.82 ± 0.11	0.77 ± 0.17	0.274	**—**	**—**	**—**
AP	0.92 ± 0.05	0.93 ± 0.04	0.98 ± 0.02	**0.000**	**0.015**	**0.000**	**0.000**
Acoustic parameters				Acoustic parameters			
HNR_audio_	–3.8 ± 1.8	2.35 ± 4.70	7.87 ± 3.87	**0.000**	**0.000**	**0.000**	**0.000**
NNE_audio_	–5.9 ± 1.3	–9.69 ± 4.55	–15.28 ± 3.66	**0.000**	**0.000**	**0.000**	**0.000**
CPP_audio_	15.0 ± 1.2	16.8 ± 2.3	19.6 ± 3.8	**0.000**	**0.000**	**0.000**	**0.000**
Shim%_audio_	16.8 ± 4.3	11.6 ± 5.9	5.5 ± 2.9	**0.000**	**0.000**	**0.000**	**0.000**
Jitt%_audio_	16.2 ± 3.9	8.3 ± 4.1	4.3 ± 3.7	**0.000**	**0.000**	**0.000**	**0.000**
Subglottal pressure parameters				Subglottal pressure parameters			
HNR_*P*__sub_	3.1 ± 3.1	3.9 ± 5.0	12.0 ± 6.1	**0.000**	0.719	**0.000**	**0.000**
NNE_*P*__sub_	–11.8 ± 5.5	–10.5 ± 5.0	–18.2 ± 5.5	**0.000**	0.151	**0.000**	**0.000**
CPP_P__sub_	14.3 ± 1.2	16.0 ± 2.1	17.7 ± 3.3	**0.000**	**0.000**	**0.000**	**0.000**
Shim%_*P*__sub_	12.2 ± 5.9	9.6 ± 5.9	2.7 ± 2.8	**0.000**	0.020	**0.000**	**0.000**
Jitt%_*P*__sub_	14.9 ± 4.6	10.4 ± 6.4	4.4 ± 4.5	**0.000**	**0.000**	**0.000**	**0.000**
